# ^13^C-Stable isotope resolved metabolomics uncovers dynamic biochemical landscape of gut microbiome-host organ communications in mice

**DOI:** 10.1186/s40168-024-01808-x

**Published:** 2024-05-15

**Authors:** Xia Xiao, Yixuan Zhou, Xinwei Li, Jing Jin, Jerika Durham, Zifan Ye, Yipeng Wang, Bernhard Hennig, Pan Deng

**Affiliations:** 1 Jiangsu Key Laboratory of Neuropsychiatric Diseases and College of Pharmaceutical Sciences, Soochow University, 199 Ren-Ai Road, 1132 Yunxuan Bldg, Suzhou, 215123 China; 2 Superfund Research Center, University of Kentucky, Lexington, KY USA; 3 Department of Toxicology and Cancer Biology, College of Medicine, University of Kentucky, Lexington, KY USA; 4 Department of Biopharmaceutical Sciences, College of Pharmaceutical Sciences, Soochow University, Suzhou, China; 5 Department of Animal and Food Sciences, Martin-Gatton College of Agriculture, Food and Environment, University of Kentucky, 900 S. Limestone St, 501 Wethington Health Sciences Bldg, Lexington, KY 40536 USA

**Keywords:** Microbiome, Metabolomics, Stable isotope, Metabolite, Inulin

## Abstract

**Background:**

Gut microbiome metabolites are important modulators of host health and disease. However, the overall metabolic potential of the gut microbiome and interactions with the host organs have been underexplored.

**Results:**

Using stable isotope resolved metabolomics (SIRM) in mice orally gavaged with ^13^C-inulin (a tracer), we first observed dynamic enrichment of ^13^C-metabolites in cecum contents in the amino acids and short-chain fatty acid metabolism pathways. ^13^C labeled metabolites were subsequently profiled comparatively in plasma, liver, brain, and skeletal muscle collected at 6, 12, and 24 h after the tracer administration. Organ-specific and time-dependent ^13^C metabolite enrichments were observed. Carbons from the gut microbiome were preferably incorporated into choline metabolism and the glutamine-glutamate/GABA cycle in the liver and brain, respectively. A sex difference in ^13^C-lactate enrichment was observed in skeletal muscle, which highlights the sex effect on the interplay between gut microbiome and host organs. Choline was identified as an interorgan metabolite derived from the gut microbiome and fed the lipogenesis of phosphatidylcholine and lysophosphatidylcholine in host organs. In vitro and in silico studies revealed the de novo synthesis of choline in the human gut microbiome via the ethanolamine pathway, and *Enterococcus faecalis* was identified as a major choline synthesis species. These results revealed a previously underappreciated role for gut microorganisms in choline biosynthesis.

**Conclusions:**

Multicompartmental SIRM analyses provided new insights into the current understanding of dynamic interorgan metabolite transport between the gut microbiome and host at the whole-body level in mice. Moreover, this study singled out microbiota-derived metabolites that are potentially involved in the gut-liver, gut-brain, and gut-skeletal muscle axes.

Video Abstract

**Supplementary Information:**

The online version contains supplementary material available at 10.1186/s40168-024-01808-x.

## Introduction

Gut microbial metabolites are known to modulate trans-kingdom interactions that affect host health and disease incidence [1]. Recent studies have indicated links between disturbances in the gut microbiome and diseases that not only affect the gut but also distal organs, such as brain [2], liver [3], and muscle [4]. An important role of the human gut microbiome is the breakdown of complex carbohydrates, and short-chain fatty acids (SCFAs) are the end products that are speculated to play a key role in the gut microbiota-host organ axes [2–5]. However, only a minor fraction of colon-derived SCFAs reach systemic circulation and peripheral tissues (9 and 2% for propionate and butyrate, respectively) [6], therefore raising questions about the impact of colon-derived SCFAs on distal organs. Moreover, although SCFAs can modulate brain functions [5], the brain uptake of SCFAs appears to be minimal, with 0.006% of the injected dose for butyrate [6]. Therefore, there remains a large gap in the understanding of gut microbiome-host tissue interactions mediated by metabolites.

Stable isotope resolved metabolomics (SIRM) enables the dynamic tracking of individual atoms through metabolic networks [7]. Our group previously used ^13^C-glycans (inulin and cellulose) as tracers to investigate the metabolic functions of the mouse fecal microbiome. In addition, SIRM has revealed fundamental features of host metabolism in mouse models after ^13^C_6_-glucose feeding [8]. Furthermore, these findings have provided important insights into nutrient preferences in microbes [9] and biochemical distributions in host organs [6]. Based on the knowledge that metabolites synthesized from the gut microbiome are essential for maintaining host metabolic homoeostasis, we endeavored to identify these metabolites using SIRM and a mouse model, and to determine whether they transit to the liver, brain, and skeletal muscle tissues.

Here, we used ^13^C-fully labeled inulin as a tracer and applied liquid chromatography-high resolution mass spectrometry (LC-HRMS) for the untargeted SIRM analysis of cecum contents and host organs (plasma, liver, brain, and skeletal muscle) at different time points after oral administration of [U-^13^C]-inulin to mice. We also applied this method in vitro to screen for the microbial producers of microbial-host communicating metabolites. These findings underline the importance of the gut microbial metabolic potentials and provide novel insights into the dynamic metabolic interactions of the gut microbiome and multiple host organs.

## Methods

### Materials

[U-^13^C]-inulin (from chicory, ≥ 97 atom % ^13^C), nonlabeled inulin (from chicory), the standards of glucose, fructose, sucrose, and 1-ketose were obtained from Sigma‒Aldrich (St. Louis, MO, USA). The authentic standard of inulin (Raftiline®) was purchased from Beneo-Orafti (Tienen, Belgium). Acetonitrile, methanol, and water (all LC/MS grade) were purchased from Fisher Scientific (Waltham, MA, USA) or Millipore Sigma (St. Louis, MO, USA). Ammonium bicarbonate and ammonium hydroxide (eluent additives for LC/MS) as well as methyl tertiary-butyl ether (MTBE) were obtained from Millipore Sigma (St. Louis, MO, USA). The IROA Mass Spectrometry Library of Standards (Sigma‒Aldrich, Darmstadt, Germany) in 96-well plate format (5 μg per well) were used. The contents of each well were dissolved according to the protocol from IROA, to yield a final concentration of 100 μg/mL. The plates were then sonicated for 5 min. Mixtures of up to 12 compounds with distinct exact masses were obtained by pooling 20 μL from each well. Spilfyter hands-in-bags were purchased from NPS Corp. (Cudahy, WI, USA), and Hungate tubes were purchased from Beijing Keenbao Biotechnology Co., Ltd (Beijing, China).

### Analysis of [U-^13^C]-inulin

The degree of polymerization (DP) of [U-^13^C]-inulin was analyzed by using high-performance anion exchange chromatography (HPAEC) coupled with pulsed amperometric detection (Thermofisher ICS-5000) with a Dionex CarboPac PA200 guard (3 × 50 mm) and analytical (3 × 250 mm) columns (Thermo Scientific, Sunnyvale, CA, USA) at 30 °C. Mobile phase A was 0.1 M sodium hydroxide solution, and mobile phase B was 0.95 M sodium acetate in 0.1 M sodium hydroxide. The samples were eluted with a linear gradient from 0% B to 44.2% B over 38 min, maintained at 44.2% B for 7 min, increased to 100% B over 3 min, and then re-equilibrated with 0% B for 10 min. The sample injection volume was 25 μL. Authentic standards of glucose, fructose, sucrose, 1-ketose, and inulin (Raftiline®, DP 3 to 60) were used to confirm the identities of mono- and poly-saccharides in [U-^13^C]-inulin.

### Animal experiment

Wild type male and female C57BL/6 mice (8 weeks old) were obtained from the Jackson Laboratory (Bar Harbor, ME, USA). The mice were allowed access to water and a standard chow diet (2918 Teklad Irradiated Global 18% Protein Rodent Diet), which contained 18.4% protein, 6% fat, 44.3% carbohydrate, and 18.5% fiber. The mice were maintained at 23 °C with a 14/10-h light–dark cycle. After acclimatization for 1 week, the mice (weighing 22.9 ± 3.3 g by the day of inulin oral gavage) were randomly divided into four groups (*n* = 3/group/sex) and singly housed in metabolic cages (Tecniplast, West Chester, PA, USA). Dosing suspensions of [U-^13^C]-inulin and nonlabeled inulin were prepared in reverse osmosis water, and the final concentration was achieved at 0.5 mg/μL. Because of the fairly aqueous solubility of inulin [10], the dosing solution was provided as a slurry, and the mixture was vortexed thoroughly before administration to ensure homogeneity. After an overnight fast (8 h), the mice in groups #1–3 received a single oral dose of [U-^13^C]-inulin suspension at 10 μL/g by gavage. The mice in group #4 (control, *n* = 3/sex) were orally gavaged with the same dose of nonlabeled inulin suspension. Cecum contents, plasma, liver, brain, and skeletal muscle tissues (collected from the hindlimbs of the mice) were harvested at 6 h (mice in group #1), 12 h (mice in group #2), and 24 h (mice in groups #3 and #4) after [U-^13^C]-inulin or non-labeled inulin administration. The samples were snap-frozen and stored in a − 80 °C freezer before analysis. All animal procedures were approved by the Institutional Animal Care & Use Committee (IACUC) of the University of Kentucky under study protocol number 2020–3561.

### Sample extraction

The cecum content, liver, muscle, brain, and plasma samples were extracted and analyzed using methods we reported previously [11, 12]. Briefly, aliquoted tissue samples (~ 25 mg of liver, brain and muscle and ~ 50 mg of cecum content) were homogenized in 200 μL of 0.1% ammonium formate. The homogenate was mixed with 1.47 mL of methanol and 5 mL of MTBE. After shaking for 30 min at room temperature, phase separation was induced by adding 1.25 mL of H_2_O, followed by centrifugation. The upper lipid phase was collected and dried under nitrogen flow, and the lower aqueous (polar) phase was pooled and lyophilized. Twenty microliters of plasma sample was extracted using 500 μL of MTBE and the extract was processed as described above. For LC‒MS analysis, the lipid residue was dissolved in 400 μL of a mixture of chloroform and methanol (2:1, v/v), and the polar residue was resuspended in 200 μL of a mixture of acetonitrile and water (4:1, v/v).

### Anaerobic incubations

Fecal samples were collected from three healthy participants who were between the age range 20–30 years old (two women and one man). None of the donors received antibiotics in the 3 months prior to sampling, and they had not suffered from any illness 6 months prior. Written consent was obtained from the donors, and all procedures involving human subjects were approved by the Soochow University Research Ethics Board under number 2022–001. Fecal microbial cells were collected as previously reported [11]. The fresh fecal sample (approximately 10 g) was blenderized in 30 mL of culture media using a glass stirring rod until a homogenous texture was achieved. The suspensions were subjected to low-speed centrifugation (500* g*, 8 min) to remove larger particles of undigested material. The supernatants were then collected and centrifuged at 3000* g* for 10 min to pellet the microbes. The microbial cells were then suspended in 2 mL of culture medium in Hungate tubes, after which ^13^C-inulin was added aseptically to achieve a final concentration of 2 mg/mL. After incubating at 37 °C for 24 h, the samples were centrifuged (3000* g*, 5 min) to collect the supernatant (culture medium). Each pellet was washed with 1 mL of fresh culture medium and centrifuged to collect the microbial cells. All procedures were performed under anaerobic conditions (with the Spilfyter hands-in-bag). The microbial cells were quenched using 450 μL cold methanol immediately after collection. After brief agitation by vortexing, the samples were transferred to glass tubes. Then, 5 mL of MTBE was added to each tube, and the polar as well as lipid fractions were collected as described above. The collected medium samples were lyophilized. The dried powder was stored at − 80 °C before analysis.

### In silico and in vitro screening of choline-producing bacteria

The metabolic capacity of the gut microbiome species was compared using MetaCyc (https://metacyc.org/). The organism database of seven representative strains that are capable of metabolizing carbohydrates was selected, namely, *Akkermansia muciniphila* ATCC BAA-835 [13], *Bacteroides fragilis* ATCC25285, *Bacteroides thetaiotaomicron* VPI-5482 [14], *Bacteroides ovatus* ATCC 8483 [15], *Bifidobacterium adolescentis* P2P3 [16], *E. faecalis* ATCC 29200 [17], and *Faecalibacterium prausnitzii* A2-165 [18]. A comparative analysis of metabolic pathways was performed using the SmartTables function (option: pathways; https://metacyc.org/comp-genomics?tables=pathway&orgids=%28AMUC349741+BFRA272559+GCF_000154125-HMP+GCF_000011065+GCF_003856735+GCF_000159655+GCF_002734145%29). The total number of pathways associated with carbohydrate degradation in each species was calculated using Microsoft Excel.

To identify potential choline-producing bacteria, enzymes involved in the serine-ethanolamine pathways associated with choline production (phosphoethanolamine/phosphocholine phosphatase, choline monooxygenase, choline dehydrogenase, choline oxidase, serine decarboxylase, ethanolamine kinase, phosphoethanolamine N-methyltransferase, and phospholipase D) were identified from the MetaCyc database and the literature [19]. The query enzyme sequences were manually collected from the RefSeq database of the NCBI and from the KEGG database (Supplementary Data 1). A BLASTP search was performed by applying a cutoff of 45% for sequence identity along with an e-value threshold of 1 × 10^−5^. RefSeq data from 200 randomly selected healthy volunteers in the Human Microbiome Project (HMP) database were used for the calculation of the number of models and relative abundance of microbial genera. All the sequences were compared against reference sequences from the HMP database (https://www.hmpdacc.org, accessed on 12 July 2023).

Considering the abundance of bacterial species in the human gut, *B. fragilis* (ATCC25285), *B. thetaiotaomicron* (ATCC 29148), and *E. faecalis* (ATCC29212) were used for the screening of bacterial strains that can metabolize ^13^C-inulin. The bacterial lyophilized powder was brought into an anaerobic glove bag and grown in the following media: GAM (Gifu Anaerobic Medium, Hope Bio-Technology Co., Ltd., Qingdao, China) for *B. fragilis* and *B. thetaiotaomicron* and LB (Luria Broth, prepared as previously reported) for *E. faecalis*. A single colony was transferred to a Hungate tube containing the aforementioned liquid medium and incubated with [U-^13^C]-inulin at 2 mg/mL. The samples were collected at the late log phase of bacterial growth as determined by the absorbance at 600 nm (OD_600_). Each experiment was performed in triplicate.

### LC-HRMS methods

Stable isotope resolved metabolomic and lipidomic analyses were performed using either a Q Exactive or a Q Exactive Focus Orbitrap mass spectrometer, both equipped with an Ion Max API source and a HESI II probe, and were coupled to a Dionex UltiMate 3000 UHPLC system (Thermo Fisher Scientific).

Chromatographic separation for metabolome analysis was achieved using a Sequant ZIC-pHILIC column (2.1 × 150 mm, 5 μm) (Merck). Buffer A was composed of 20 mM ammonium carbonate and 0.1% ammonium hydroxide, and buffer B was acetonitrile. The chromatographic gradient was run at a flow rate of 150 μL/min as follows: 0–20 min, linear gradient from 80 to 20% B; 20–21 min, hold at 20% B min; 21–22 min, linear gradient to 80% B; 22–28 min, re-equilibrate at 80% B. The mass spectrometer was operated in full-scan, positive and negative modes with the spray voltage set to 3.0 kV (+ / −), the heated capillary held at 275 °C, and the HESI probe held at 350 °C. The sheath gas flow was set to 40 units, the auxiliary gas flow was set to 15 units, and the sweep gas flow was set to 1 unit. The MS data acquisition was performed in the range of 59–850 m/z, with the resolution set at 70,000, the AGC target at 10^6^, and the maximum injection time at 100 ms.

Lipid extracts were separated on a Waters ACQUITY BEH C8 column (2.1 × 100 mm, 1.7 μm), and the temperature was maintained at 40 °C. The flow rate was 250 μL/min, and the mobile phases consisted of 60:40 water/acetonitrile (A), and 90:10 isopropanol/acetonitrile (B), both of which contained 10 mM ammonium formate and 0.1% formic acid. The samples were eluted with a linear gradient from 32% B to 97% B over 25 min, maintained at 97% B for 4 min and re-equilibrated with 32% B for 6 min. The sample injection volume was 5 μL. The mass spectrometer was operated in positive and negative ionization modes. The full scan and fragment spectra were collected at resolutions of 70,000 and 17,500, respectively.

### Metabolite identification

The LC‒MS raw files were first converted to.abf files with the Reifycs ABF converter (http://www.reifycs.com/AbfConverter/index.html) for compatibility with MS-DIAL. LC‒MS data were processed with MS-DIAL version 4.80. The mass accuracy was set to 0.01 and 0.025 Da for the MS1 and MS2 tolerances, respectively. For peak detection, a minimum peak height of 3 × 10^4^ was used, and the mass slice width was 0.1. Peaks were aligned on the sample from group #4 (nonlabeled inulin treatment) with a RT tolerance of 0.2 min and a mass tolerance of 0.015 Da. For metabolomics analysis, the features were identified using “MSMS-Public-POS-VS15” and “MSMS-Public-NEG-VS15” as MSP files for positive and negative ionization data, respectively. For lipidomic analysis, HCOOCH_4_ was selected as the main ion, and MS-DIAL formulates mass spectral fragmentations of lipids across 117 lipid subclasses were selected. The MS1 and MS2 accurate mass tolerances were set to 0.01 and 0.05 Da, respectively. ^13^C isotope tracking was performed using the group #4 sample as the nonlabeled reference file. The metabolites were further confirmed by comparison of their ion features with those of the IROA reference library of authentic chemical standards, which included retention times, precursor ions, and their associated product ion mass spectra.

### Data processing, statistics, and visualization

The LC‒MS peak areas of the metabolites and isotopologues were integrated and exported to Excel via the MS-DIAL (version 4.80). The fractional ^13^C enrichment in metabolites was obtained after natural abundance stripping using Escher-Trace [20], and the ^13^C fraction of each isotopologue was calculated using the protocol described previously [8, 11]. Time-dependent changes in the metabolome of mouse cecum contents were analyzed using the MetATT and MEBA plug-ins from MetaboAnalyst 5.0 (https://www.metaboanalyst.ca). A list of variables that changed over time was ranked by Hotelling’s *T*^2^. Principal component analysis (PCA) was used to identify initial trends and clusters in the datasets, which was performed using the MetaboAnalyst 5.0 web portal (www.metaboanalyst.ca). Three- and two-way clustered (time, sex, inulin treatment) heatmaps were constructed using Metaboanalyst. A quantitative metabolite set enrichment analysis (qMSEA) was conducted on the altered metabolites using their PubChem CID identifiers and log-transformed peak area pairs as the data input for computation based on a priori defined sets of metabolites in pathway-associated metabolite sets; metabolite sets that contained at least two compounds were used.

Statistical analyses of the metabolite data were performed using the GraphPad Prism version 7.04 for Windows (GraphPad Software Inc., La Jolla, CA, USA). Student’s *t* test was applied for paired groups. The *p* values (all analyses) were accepted as statistically significant at *p* < 0.05.

## Results

### Time-dependent metabolism of ^13^C-inulin in the mouse gut microbiome

**Fig. 1:**
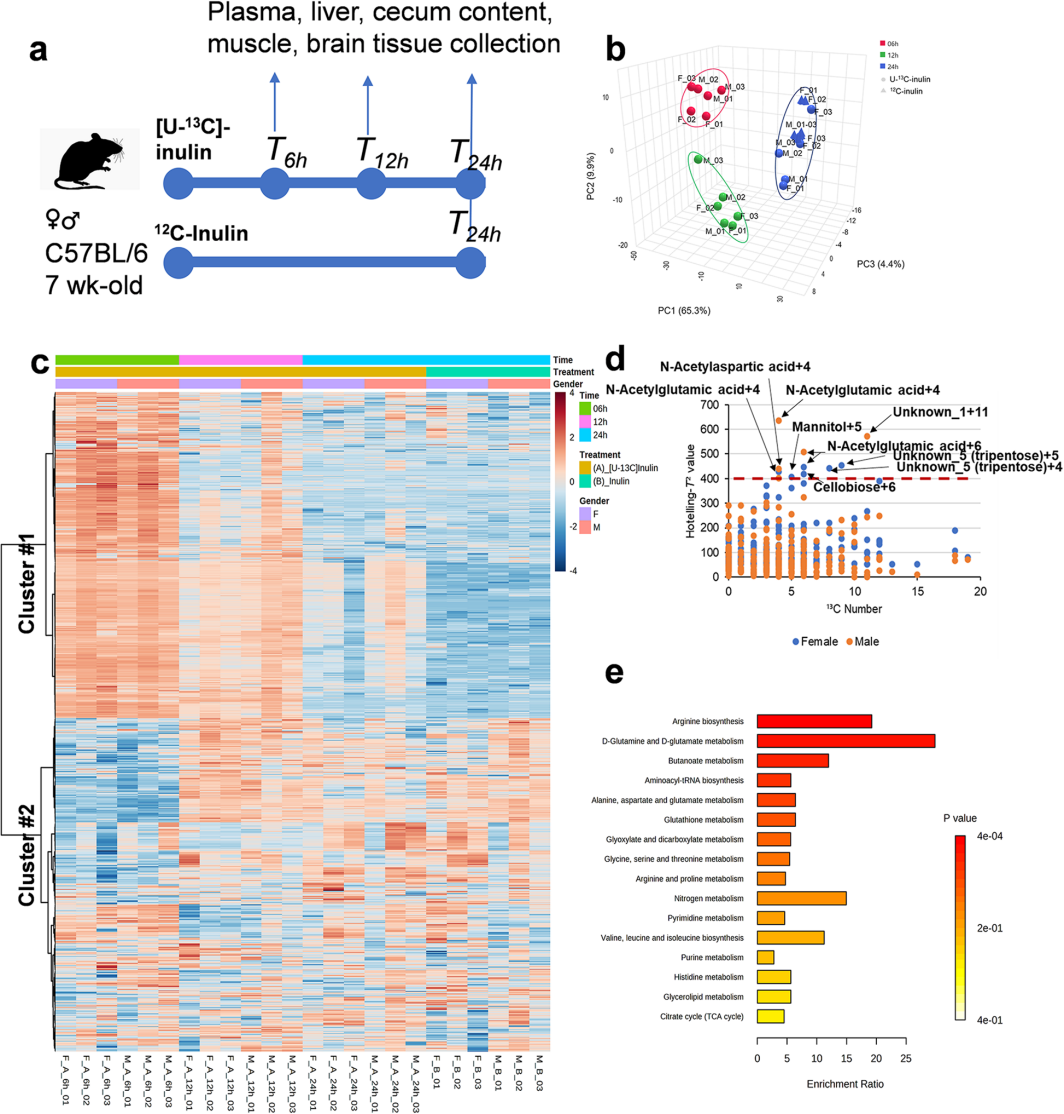
Time-depended changes of the mouse cecum content metabolome after [U-^13^C]-inulin administration. **a** Experimental in vivo characterization of the mouse metabolome after inulin administration. Male and female mice were orally administered [U-^13^C]-inulin or ^12^C-inulin (*n* = 3/time point/sex) at 5 mg/g. Biological samples were collected at 6, 12, and 24 h after [U-^13^C]-inulin administration and at 24 h after ^12^C-inulin administration. **b** Multivariate unsupervised principal component analysis shows the discrimination of biochemicals between [U-^13^C]-inulin (circles, red: *T*_6h_, green: *T*_12h,_ blue: *T*_24h_) and ^12^C-inulin (triangles, blue: *T*_24h_) treated mice. **c** Heatmap indicates the apparent time-dependent effects of [U-^13^C]-inulin on the cecum content metabolome. **d** Multivariate empirical Bayes time-series analysis of the cecum content metabolome after [U-^13^C]-inulin administration. Biochemicals with high Hotelling’s *T*^2^ values (> 400) comprise those with the number of ^13^C ranged from 4 to 11. **e** Pathway analysis of metabolites that were inversely correlated with time, regardless of sex, revealed that these metabolites were mainly enriched in the amino acid and short-chain fatty acid metabolism pathways

The DP value of [U-^13^C]-inulin was analyzed by the HPAEC-PAD method (Supplementary Fig. 1), and polysaccharides with a DP ≥ 3 accounted for 87.4% of the [U-^13^C]-inulin content based on the peak areas. After oral gavage of [U-^13^C]-inulin, a total number of 1172 biochemicals were detected in the cecum contents of the mice, including 358 metabolites and their ^13^C isotopologues. These metabolites were assigned to the pathways of sugar metabolism, amino acid biosynthesis, metabolism of organic acids, nucleotide biosynthesis, coenzyme biosynthesis, and central carbon metabolism. Multivariate unsupervised PCA was performed to discriminate biochemicals between [U-^13^C]-inulin (*T*_6h_, *T*_12h,_ and *T*_24h_) and ^12^C-inulin (*T*_24h_) treated mice (i.e., nonlabeled control samples) (Fig. 1a, b). The principal component PC1 captured 65.3% of the total variation from the PCA models based on the 1172 biochemicals. Clear separations were observed among samples collected at different time points after [U-^13^C]-inulin administration from both male and female mice (Fig. 1b). Patterns of metabolite abundance in the heatmap of the cecum content metabolome indicated apparent time-dependent impacts of [U-^13^C]-inulin (Fig. 1c). In cluster #1 of the heatmap, the abundance of biochemicals was inversely correlated with the time and was the highest in the *T*_6h_ samples. In addition, the biochemicals in cluster #1 were lower in ^12^C-inulin-treated mice than in ^13^C-inulin-treated mice. In contrast, for biochemicals in the cluster #2, comparable abundances were observed among the ^13^C-inulin (*T*_12h_ and *T*_24h_) and ^12^C-inulin groups, suggesting that the biochemicals in cluster #2 were features associated with dietary intake. These results suggested that biochemicals uniquely related to [U-^13^C]-inulin administration were mainly in cluster #1 of the heatmap, and the abundance of which decreased with the time.

**Fig. 2:**
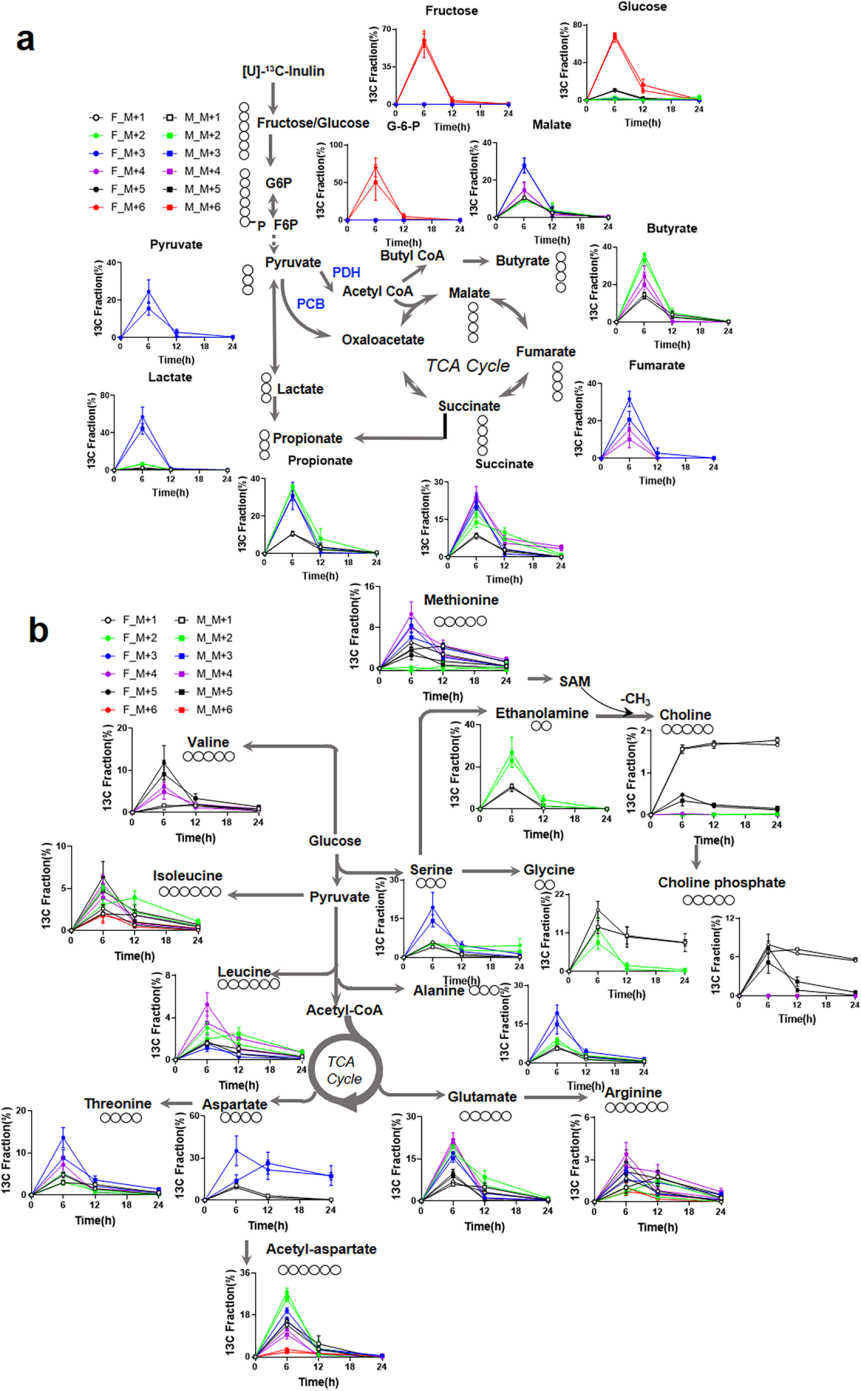
^13^C fractional enrichment of metabolites in cecum contents after [U-^13^C]-inulin administration. Mice of each sex (F: female, M: male) were orally administered [U-^13^C]-inulin (*n* = 3/time point/gender) at 5 mg/g, and cecum contents were collected at 6, 12, and 24 h after administration. The samples were processed and analyzed by LC‒HRMS as described in the “Methods” section. Tracing of inulin carbon through the **a** central carbon metabolism pathway and **b** amino acid metabolism pathway. The circles indicate the carbon on each metabolite. The values shown are the mean ± SEM (*n* = 3)

Two different approaches were used to further analyze the data: (1) Multivariate empirical Bayes time-series analysis (MEBA) was used to determine the time-course metabolome profiles in the cecum contents of male and female mice separately. Biochemicals with high Hotelling’s *T*^2^ values (> 400) included those with ^13^C numbers ranging from 4 to 11 (Fig. 1d); (2) Correlation analysis was performed to identify metabolites whose abundance decreased with time, regardless of sex. A total of 75 biochemicals were identified with correlation factors ranging from − 0.90 to − 0.97 (Supplementary Data 2). It is important to note that these biochemicals were all ^13^C-labeled metabolites, including sugars, short-chain fatty acids, nucleotides, and amino acids and derivatives. In addition, five biochemicals that were not assigned to metabolites in the database were identified. Among those, the biochemicals identified in the MEBA were also detected, including N-acetylglutamate, cellobiose, mannitol, unknown_1, and unknown_5 (Fig. 1d; Supplementary Data 2). Based on the precursor and product ion information from high-resolution mass spectrometry, unknown_5 was proposed to be a tripentose with a molecular formula of C_15_H_27_O_13_ (Supplementary Fig. 2). Pathway analysis of these metabolites revealed that they were enriched in pathways related to amino acid metabolism and short-chain fatty acid metabolism (Fig. 1e).

We then analyzed the ^13^C fractional enrichment of metabolites related to central carbon metabolism (Fig. 2a) and amino acid metabolism (Fig. 2b). A considerable abundance of ^13^C_6_ isotopologues in glucose, fructose, and hexose-6-phosphate (Fig. 2a) was evident in both male and female mice at 6 h after [U-^13^C]-inulin administration. The ^13^C labels of central carbon metabolites significantly diminished in the *T*_12h_ and *T*_24h_ samples (Fig. 2a), suggesting further and complete utilization of these metabolites by either the gut microbiome or the host. As the gut microbiome is known to be active in gluconeogenesis and through the pentose phosphate pathway (PPP)^12^, mixed ^13^C labeling patterns observed for glucose were expected, and the ^13^C mixing of glucose (^13^C_5_,^13^C_2_ and ^13^C_3_ isotopologues) in the cecum contents could arise from gluconeogenesis and/or the non-oxidative branch of the PPP. The ^13^C mixing of Krebs cycle metabolites (e.g., ^13^C_1_ -^13^C_4_ isotopologues of succinate, fumarate, and malate) was also detected in the cecum contents (Fig. 2a). High fractional enrichment of the ^13^C_3_ isotopologue of malate and fumarate could be generated from pathways catalyzed by pyruvate carboxylase-initiated Krebs cycle. The fully labeled ^13^C_4_ isotopologue is presumably produced from ^13^C_6_-glucose via at least 4 turns of the canonical Krebs cycle activity and/or 2 turns of the cycle activity with anaplerotic pyruvate carboxylation. We further found extensive ^13^C labeling of SCFAs, with ^13^C_2_ and ^13^C_3_ accounting for 67% of the total carbon pool for propionate, and ^13^C_2_ together with ^13^C_4_ fractions accounting for 57% of the total carbon pool for butyrate, which was consistent with the findings of our previous in vitro study^12^.

A total of fifteen ^13^C-labeled amino acids and their metabolites, including the conditionally essential amino acid arginine and essential amino acids, i.e., threonine, leucine, isoleucine, and valine, were detected in the cecum contents from ^13^C-inulin treated mice. In addition, the labeling was observed up to 24 h after [U-^13^C]-inulin administration (Fig. 2b), suggesting continuous de novo synthesis of amino acids and/or circulation of amino acids and their precursors between host organs and the gut. Leucine, isoleucine, and valine are branched-chain amino acids (BCAAs). ^13^C-labeled BCAA contributed to 9.9, 18.6, and 17.4% of the total carbon pool for leucine, isoleucine, and valine respectively, with ^13^C_4_ being the major isotopologue for leucine, and ^13^C_4_ as well as ^13^C_5_ were the major isotopologues for isoleucine and valine. The fully labeled compounds were the major isotopologues for choline (^13^C_5_), choline phosphate (^13^C_5_), ethanolamine (^13^C_2_), serine (^13^C_3_), and alanine (^13^C_3_). Considerable mixing of ^13^C labels (^13^C_2_–^13^C_6_ isotopologues) was evident for glutamate and arginine (Fig. 2b).

### ^13^C-labeled metabolites shared by the gut microbiome and host plasma

**Fig. 3:**
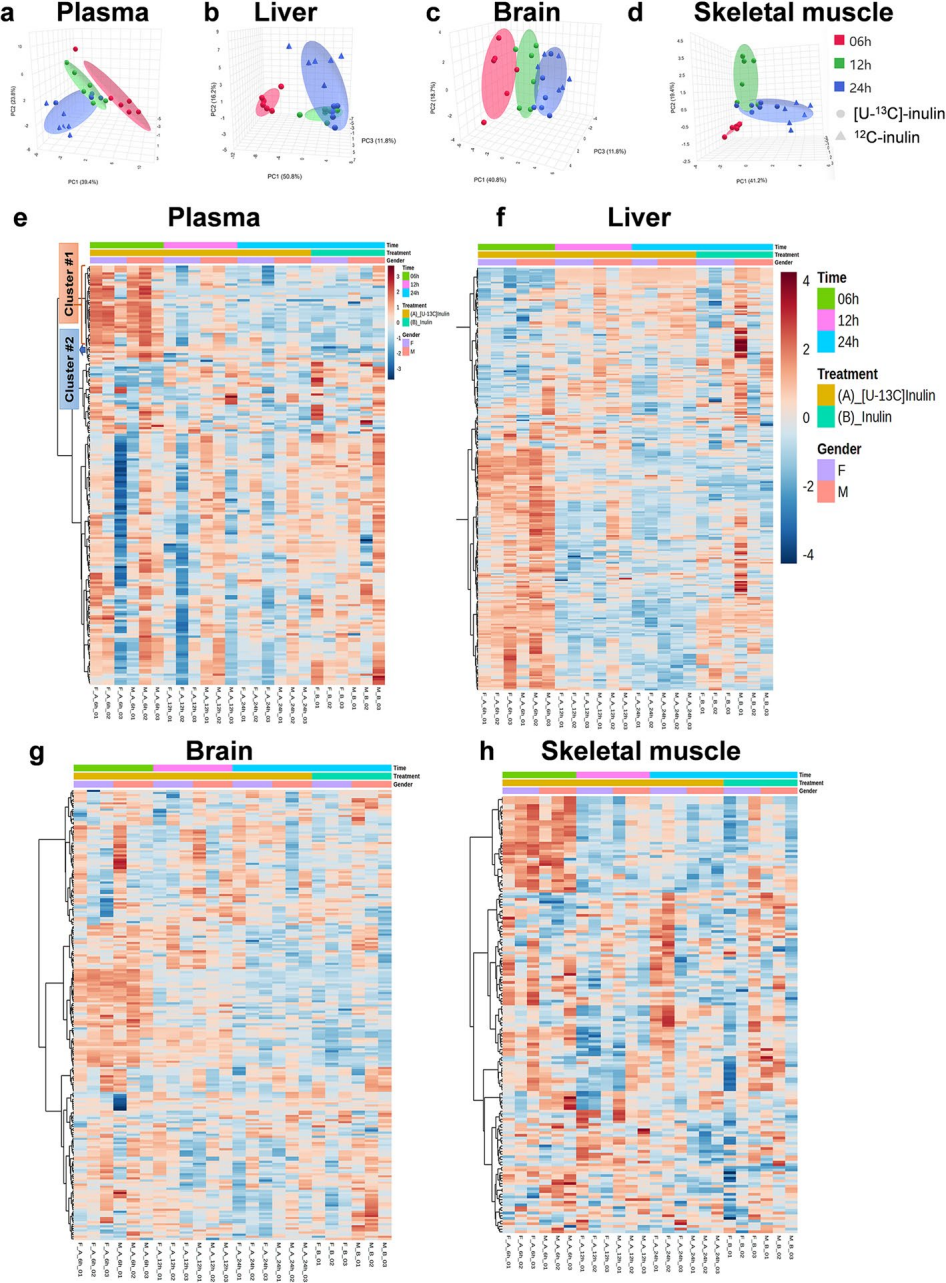
The changes in mouse plasma, liver, brain, and skeletal muscle metabolome after [U-^13^C]-inulin (*T*_6h_, *T*_12h_, and *T*_24h_) and ^12^C-inulin (*T*_24h_) administration. Mice of each sex (F: female, M: male) were orally administered [U-^13^C]-inulin or inulin (*n* = 3/time point/gender) at 5 mg/g. Biological samples were collected at 6, 12, and 24 h after [U-^13^C]-inulin administration and at 24 h after inulin administration. **a–d** PCA shows the changes in the mouse metabolome after [U-^13^C]-inulin (*T*_6h_, *T*_12h_, and *T*_24h_) and ^12^C-inulin (*T*_24h_) administration. **e,f** Heatmap analysis shows the time-dependent changes in the mouse metabolome after [U-^13^C]-inulin administration. In plasma samples, the differentially expressed biochemicals were found mainly in clusters #1 and #2

The PCA was performed to evaluate the difference in the mouse plasma metabolome after [U-^13^C]-inulin (*T*_6h_, *T*_12h,_ and *T*_24h_) and ^12^C-inulin (*T*_24h_) administration (Fig. 3a). There were clear separations among the samples collected at different time points in [U-^13^C]-inulin-treated mice, indicating time-dependent variation in the plasma biochemicals after [U-^13^C]-inulin administration. However, the *T*_24h_ samples from the [U-^13^C]-inulin and ^12^C-inulin groups overlapped with each other, suggesting that the changes in biochemicals uniquely related to [U-^13^C]-inulin were diminished at 24 h after administration. In addition, no sex differences were observed in either [U-^13^C]-inulin or ^12^C-inulin-treated mice. A heatmap analysis further confirmed the time-dependent changes in the plasma metabolome after [U-^13^C]-inulin administration (Fig. 3e), with the *T*_6h_ and *T*_12h_ samples containing the highest levels of biochemicals shown in clusters #1 and #2 in the heatmap.

To identify the plasma biochemicals that uniquely associated with [U-^13^C]-inulin, a linear model with covariate adjustment was used for data analysis: (1) Plasma metabolome data after [U-^13^C]-inulin (*T*_6h_, *T*_12h_, and *T*_24h_) administration were used to identify time-dependently changed biochemicals associated with [U-^13^C]-inulin administration. “Time” was set as the primary dependent variable, and *T*_24h_ was set as the reference group. A total number of 35 biochemicals were identified with an adjusted *p* < 0.05. Among those, 26 biochemicals were ^13^C isotopologues of 15 metabolites including amino acids, short-chain fatty acids, and their derivatives (Supplementary Data 3), which were located mainly in cluster #1 of the heatmap (Fig. 3e); (2) “Treatment” was used as the primary dependent variable to identify biochemicals that correlate with treatment ([U-^13^C]-inulin vs ^12^C-inulin) regardless of time point and sex. Three metabolites with adjusted *p* < 0.05 and LogFC (fold change) > 1.4 were identified (Supplementary Data 4), including ^13^C_5_-choline, ^13^C_5_-betaine, and ^13^C_3_-lactate; these metabolites were biochemicals in cluster #2 of the heatmap (Fig. 3e). Taken together, ^13^C isotopologues of a total number of 16 metabolites were identified in plasma, including ^13^C-labeled amino acids, SCFAs, and their derivatives, and these were also the major labeled species in the cecum contents, suggesting that they were the major metabolites derived from [U-^13^C]-inulin degraded by the gut microbiome that could be absorbed into the host systemic circulation.

### ^13^C differently incorporated into mouse liver, brain, and skeletal muscle tissues

**Fig. 4:**
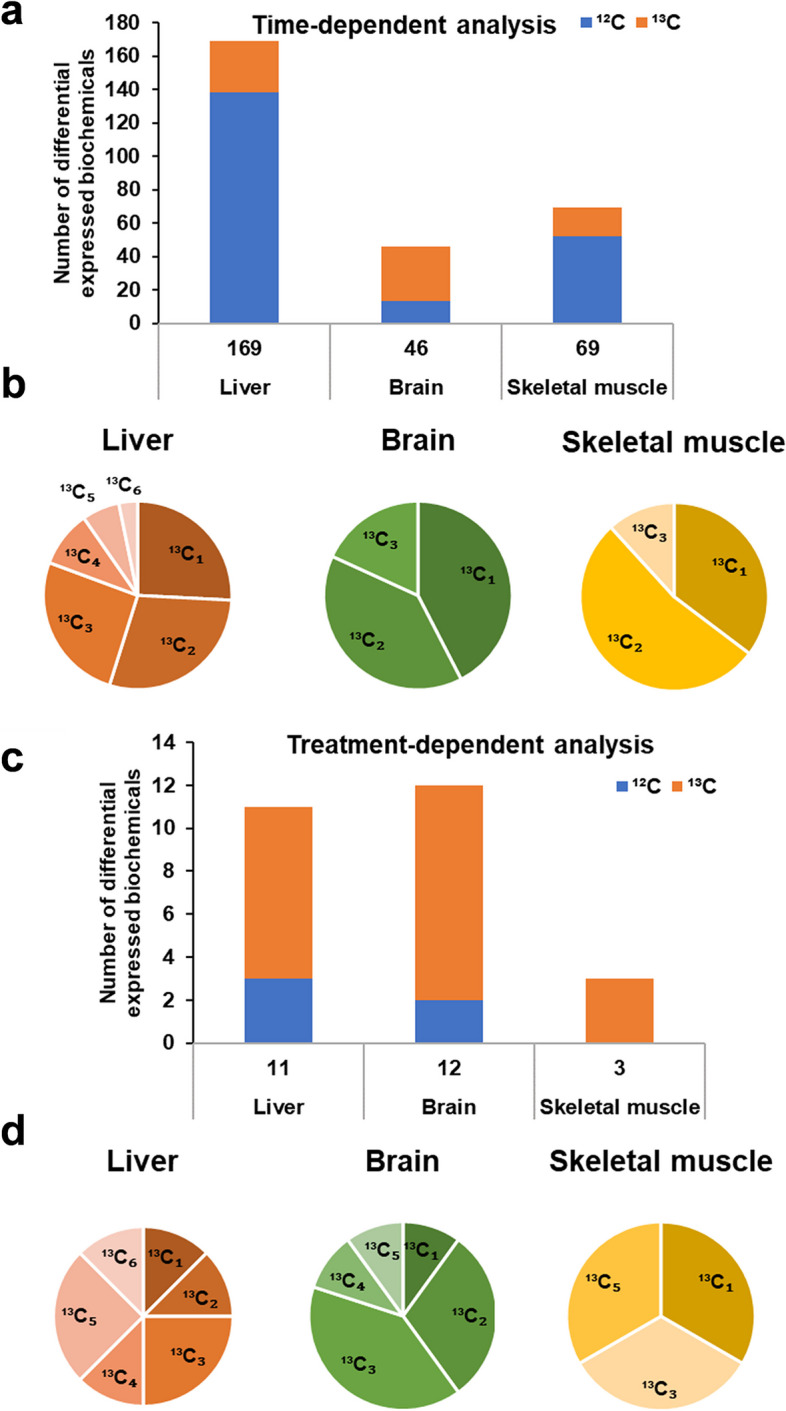
^13^C differently incorporated into mouse liver, brain, and skeletal muscle tissues. **a** Time-dependent analysis shows the number of differentially expressed biochemicals in the mouse liver, brain, and skeletal muscle after [U-^13^C]-inulin administration. The blue bar indicates the non-labeled features (^12^C), and the orange bar represents the labeled features (^13^C). **b** Relative distribution of ^13^C isotopologues for differentially expressed biochemicals (time-dependent analysis) detected in the liver, brain, and skeletal muscle. A total number of 31, 33, and 17 differentially expressed biochemicals were ^13^C isotopologues, with the number of ^13^C ranging from 1 to 6 in the liver, and from 1 to 3 in the brain and skeletal muscle. **c** Treatment-dependent analysis shows the number of differentially expressed biochemicals in the mouse liver, brain, and skeletal muscle by comparing [U-^13^C]-inulin treated and ^12^C-inulin treated groups. **d** Distribution of ^13^C isotopologues of differentially expressed biochemicals (treatment-dependent analysis) detected in the liver, brain, and skeletal muscle. A total number of 8, 10, and 3 differentially expressed biochemicals were ^13^C isotopologues, with the number of ^13^C ranging from 1 to 6 in the liver, and from 1 to 5 in the brain and skeletal muscle

**Fig. 5:**
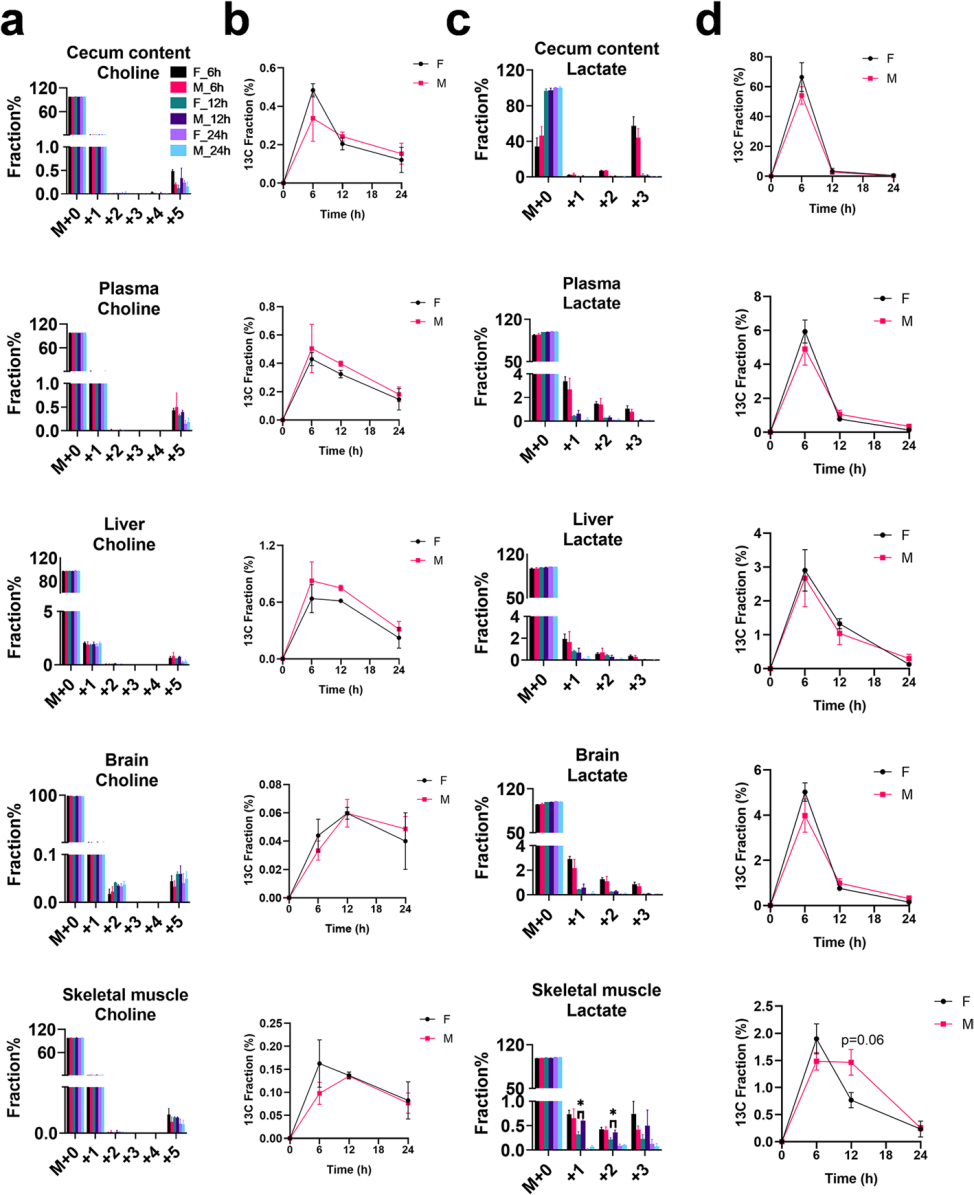
The ^13^C enrichment profile and time-dependent changes of ^13^C-labeled choline and lactate in different biological samples after oral administration of [U-^13^C]-inulin to male and female mice. **a** ^13^C fractional enrichments in the isotopologues of choline detected at different time points (6, 12, and 24 h) in cecum content, plasma, liver, brain, and skeletal muscle, respectively. The *x*-axis denotes the number of ^13^C atoms present in each compound. **b** Time-dependent changes of ^13^C_5_-choline in different samples. **c** ^13^C fractional enrichments in the isotopologues of lactate detected in different samples. **d** Time-dependent changes of ∑^13^C_n_-lactate (*n* = 1–3) in different samples. The values shown are the mean ± SEM (*n* = 3). * 0.01 < *p* < 0.05, two-tailed *t*-test (see “Methods”)

The PCA (Fig. 3b–d) and heatmap (Fig. 3f–h) analyses revealed differences in the liver, brain and skeletal muscle metabolomes of the mice after [U-^13^C]-inulin (*T*_6h_, *T*_12h_, and *T*_24h_) or ^12^C-inulin (*T*_24h_) administration. A linear model with covariate adjustment, as mentioned above, was applied to identify changes in biochemicals associated with [U-^13^C]-inulin administration in different tissues. In the time-dependent analysis of [U-^13^C]-inulin treated samples, a total number of 169, 46, and 69 differentially expressed biochemicals (adj. *p* < 0.05) were identified in the mouse liver (Supplementary Data 5), brain (Supplementary Data 6), and skeletal muscle (Supplementary Data 7), respectively, among which 31, 33, and 17 biochemicals were ^13^C isotopologues, covering 12, 16, and 9 metabolites, respectively, with the number of ^13^C ranging from 1 to 6 (Fig. 4a, b). In the treatment-dependent analysis ([U-^13^C]-inulin vs ^12^C-inulin), a total number of 11, 12, and 3 differentially expressed biochemicals were identified in the mouse liver (Supplementary Data 8), brain (Supplementary Data 9), and skeletal muscle (Supplementary Data 10), respectively; among these, 8, 10, and 3 biochemicals were ^13^C isotopologues covering 7, 5, and 3 metabolites, respectively, with ^13^C numbers ranging from 1 to 6 (Fig. 4c, d). ^13^C_5_-choline and ^13^C_3_-lactate were the common differentially expressed ^13^C isotopologues in all three organs (Supplementary Data 8, 9, and 10). Notably, the highest enrichment of ^13^C_5_-choline was observed at 12 h in the brain after [U-^13^C]-inulin administration rather than at 6 h, which was the case for tissues including the cecum content, liver, and plasma (Fig. 5a, b). In the liver, ^13^C_5_-betain, ^13^C_5_-choline, and ^13^C_4_-dimethylglycine were the top three significantly differentially expressed biochemicals (Supplementary Data 8), suggesting that choline metabolism is the major pathway in the liver that utilizes ^13^C derived from the gut microbiome. Besides ^13^C_5_-choline and ^13^C_3_-lactate, ^13^C_1_-hydroxybutyrate was a significantly differential expressed biochemical in skeletal muscle (Supplementary Data 10), indicating that ^13^C derived from the gut microbiome could be used by the skeletal muscle to produce ketone bodies, which are alternative energy substrates for muscle. The ^13^C enrichment profile of lactate in skeletal muscle was different from that in other host organs, and ^13^C scrambled isotopologues of lactate (^13^C_1_ to ^13^C_3_) were significantly more enriched in ^13^C_1_-lactate and ^13^C_2_-lactate in male mice than in female groups at 12 h after [U-^13^C]-inulin administration (Fig. 5c). The total ^13^C enrichment of lactate (∑^13^C_*n*=1–3_) peaked at 6 h and decreased thereafter in the cecum content, plasma, liver, and brain without gender difference. However, there was a trend toward higher total ^13^C enrichment of lactate in the *T*_12h_ muscle samples from male mice than in those from female mice (*p* = 0.06, Fig. 5d).

**Fig. 6:**
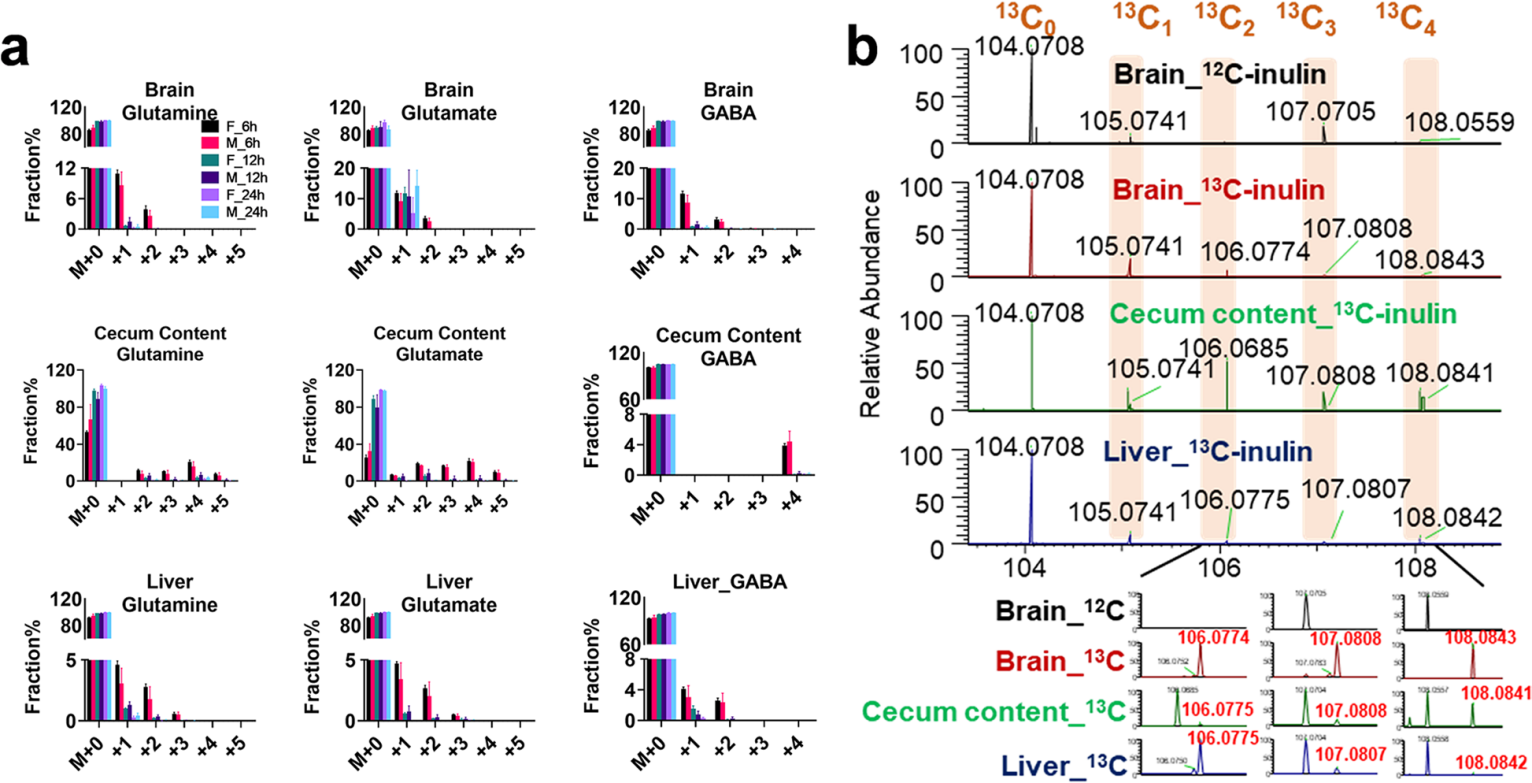
GABA in the brain can partially originate from the gut microbiome. **a** ^13^C fractional enrichments in the isotopologues of glutamine, glutamate, and GABA detected at different time points (6 h, 12 h, 24 h) in the brain, cecum content, and liver, respectively. The values shown are the mean ± SEM (*n* = 3). F: female; M: male. **b** Representative full scan mass spectra of GABA and its isotopologues (^13^C_1_–^13^C_4_) detected in the mouse brain, cecum content, and liver after oral administration of ^12^C-inulin or ^13^C-inulin. Enlarged spectra in the *m/z* range of 106–108 demonstrated the presence of a high abundance of ^13^C_4_-GABA (m/z 108.0843) in the brain and cecum contents, suggesting that gut microbiome-derived GABA partially contributes to the brain pool of GABA

In the brain, ^13^C_2_-γ-aminobutyric acid (GABA),^13^C_2_-glutamine, and ^13^C_2_-glutamate were the three most significantly differentially expressed biochemicals (adj. *P* ≤ 9.56E ^−8^), suggesting that the ^13^C derived from the gut microbiome was mainly used in the glutamine-glutamate/GABA cycle in the brain. Similar labeling patterns of glutamine, glutamate, and GABA were observed in the brain and liver (Fig. 6a). High-resolution MS/MS analysis of ^13^C_2_-glutamate suggested that the ^13^C_2_ labeling sites were on the non-terminal carbons (Supplementary Fig. 3a, b), indicating that the biosynthesis of glutamate from α-KG was catalyzed by pyruvate carboxylase, the predominant anaplerotic enzyme in the brain that regulates the neurotransmitter pools [21]. Moreover, ^13^C_4_ was one of the major isotopologues for GABA in cecum contents, and although it was presented at trace levels that the LC‒MS peak could not be integrated, the MS signal of ^13^C_4_-GABA (*m/z* 108.0843) was detected in the brain but not in the liver (Fig. 6b), suggesting that GABA in the brain could partially originate from the gut microbiome.

### Microbiota-derived choline feeds lipogenesis in the host

The major isotopologues of choline in the cecum contents and host organs were ^13^C_1_ and ^13^C_5_ (Fig. 5a), suggesting that choline could be synthesized de novo from [U-^13^C]-inulin catalyzed by the gut microbiome. Based on the labeling patterns, we propose that choline is synthesized via the ethanolamine pathway in the gut microbiome (Fig. 2b). ^13^C_1_-Choline can be generated by the ^13^C mono-methylation of unlabeled ethanolamine or by the ^12^C methylation of ^13^C_1_-ethanolamine, with S-adenosylmethionine (SAM) serving as the methyl donor, while the ^13^C tri-methylation of ^13^C_2_-ethanolamine (fully labeled) leads to the production of ^13^C_5_-choline. We found that methionine, an intermediate in transmethylation reactions and a precursor of SAM, was extensively labeled in the cecum contents, with ^13^C_1_, ^13^C_3_, ^13^C_4_, and ^13^C_5_ (fully labeled) isotopologues being detected (Fig. 2b). Therefore, ^13^C-methionine can contribute to the labeling of choline by providing the ^13^C-methyl group. In addition, both ^13^C_1_- and ^13^C_2_-ethanolamine were detected in the cecum content (Fig. 2b). These results reveal a previously underappreciated role for gut microorganisms in choline biosynthesis.

**Fig. 7:**
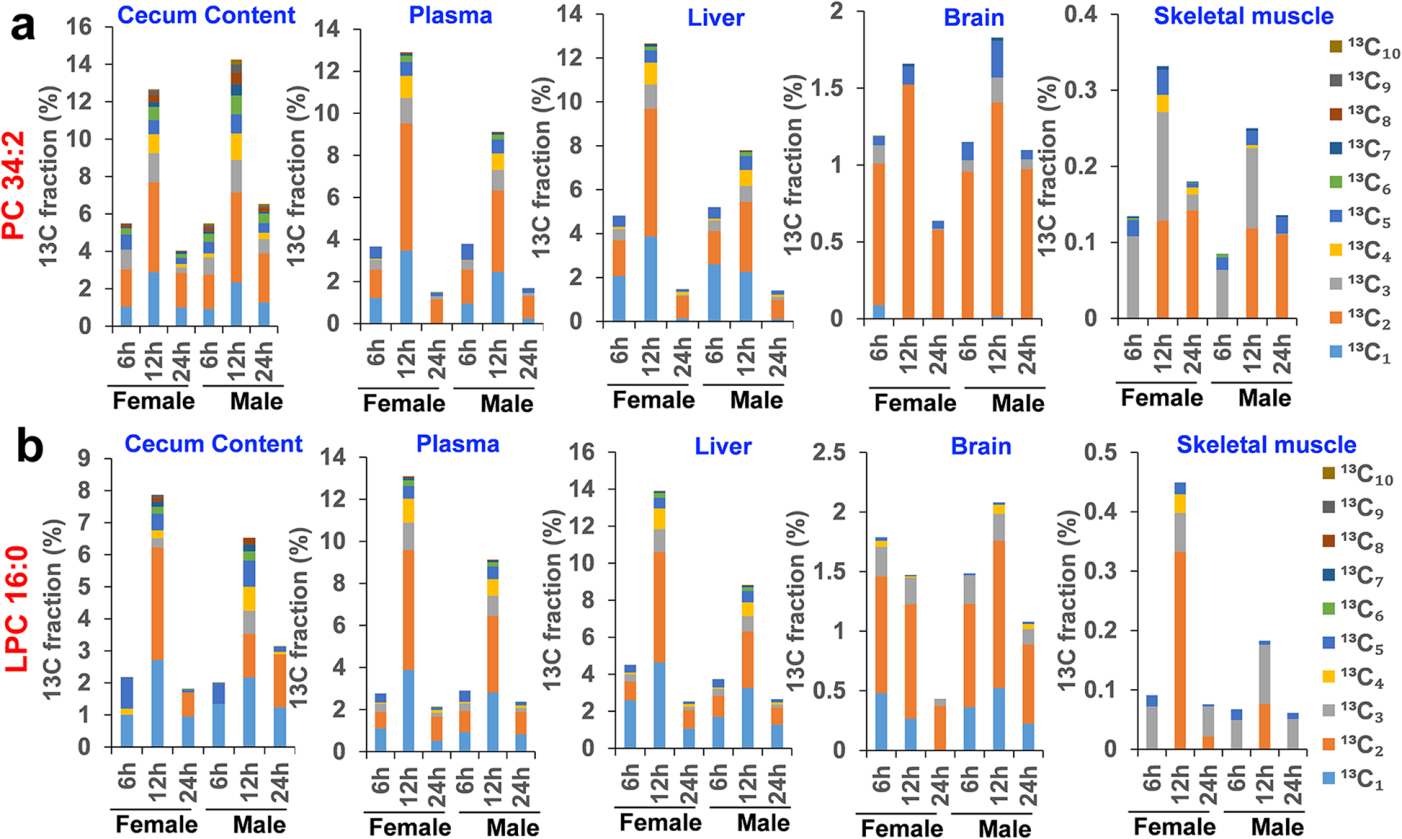
Phosphatidylcholine (PC 34:2) and lysophosphatidylcholine (LPC 16:0) are labeled with ^13^C in mice treated with [U-^13^C]-inulin. **a** ^13^C fractional enrichments in the isotopologues (^13^C_1_–^13^C_10_) of PC 34:2 detected at different time points (6, 12, and 24 h) in cecum content, plasma, liver, brain, and skeletal muscle, respectively. **b** ^13^C fractional enrichments in the isotopologues (^13^C_1_–^13^C_10_) of LPC 16:0. The average values of three biological replicates are shown

Choline can be used in the phospholipid biosynthesis. Lipidomic analysis of plasma revealed that phosphatidylcholine (PC) and lysophosphatidylcholine (LPC) were labeled with ^13^C in mice treated with [U-^13^C]-inulin (Fig. 7a, b). PC34:2 ([M + H]^+^, *m/z* 758.568) produced signals exploitable for isotopic profiling, and ten isotopologue species (^13^C_1_-^13^C_10_) were detected (Supplementary Fig. 4a). Ion dissociation spectra (ms2) analysis captured the ^13^C_5_-choline phosphate fragment ion (*m/z* 189.089) derived from ^13^C_5_- as well as ^13^C_7_- PC34:2 (Supplementary Fig. 4b), indicating the presence of ^13^C_5_-choline in the ^13^C labeled species of PC34:2. Similarly, ten isotopologues (^13^C_1_–^13^C_10_) were detected for LPC16:0 ([M + H]^+^, *m/z* 496.339; Supplementary Fig. 4c), and ms2 analysis of *m/z* 501.267 (^13^C_5_-LPC16:0) showed the fragment ion at *m/z* 189.089 (^13^C_5_-choline; Supplementary Fig. 4d), confirming that ^13^C_5_-LPC16:0 was the isotopologue with choline being fully labeled.

Time-scale analysis of lipid isotopologues revealed that the highest ^13^C labeling was achieved at 12 h after [U-^13^C]-inulin administration except for that of LPC16:0 in the brains of female mice (Fig. 7a, b). The tissues showed differences in ^13^C labeling in phospholipids. Multiple ^13^C isotopologues (^13^C_1_–^13^C_10_) were observed in the cecum content, plasma, and liver, with up to 14.2% of ^13^C fractional enrichment, suggesting that the labeling likely occurred on both the fatty acyl chains and the choline head group (^13^C_*n*_, *n* ≥ 5). In brain samples, ^13^C_1_, ^13^C_2_, ^13^C_3_, and ^13^C_5_ were the major phospholipid isotopologues, which accounted for up to 2.1% of the total carbon pool. The ^13^C fraction enrichment in phospholipids was the lowest in skeletal muscle compared with other organs, which accounted for up to 0.45% of the total carbon pool, and ^13^C_3_ was the major isotopologue species, suggesting that the labeling mainly occurred on the glycerol backbone.

### E. faecalis is the major species utilizing ^13^C-Inulin to produce ^13^C_5_-choline

**Fig. 8:**
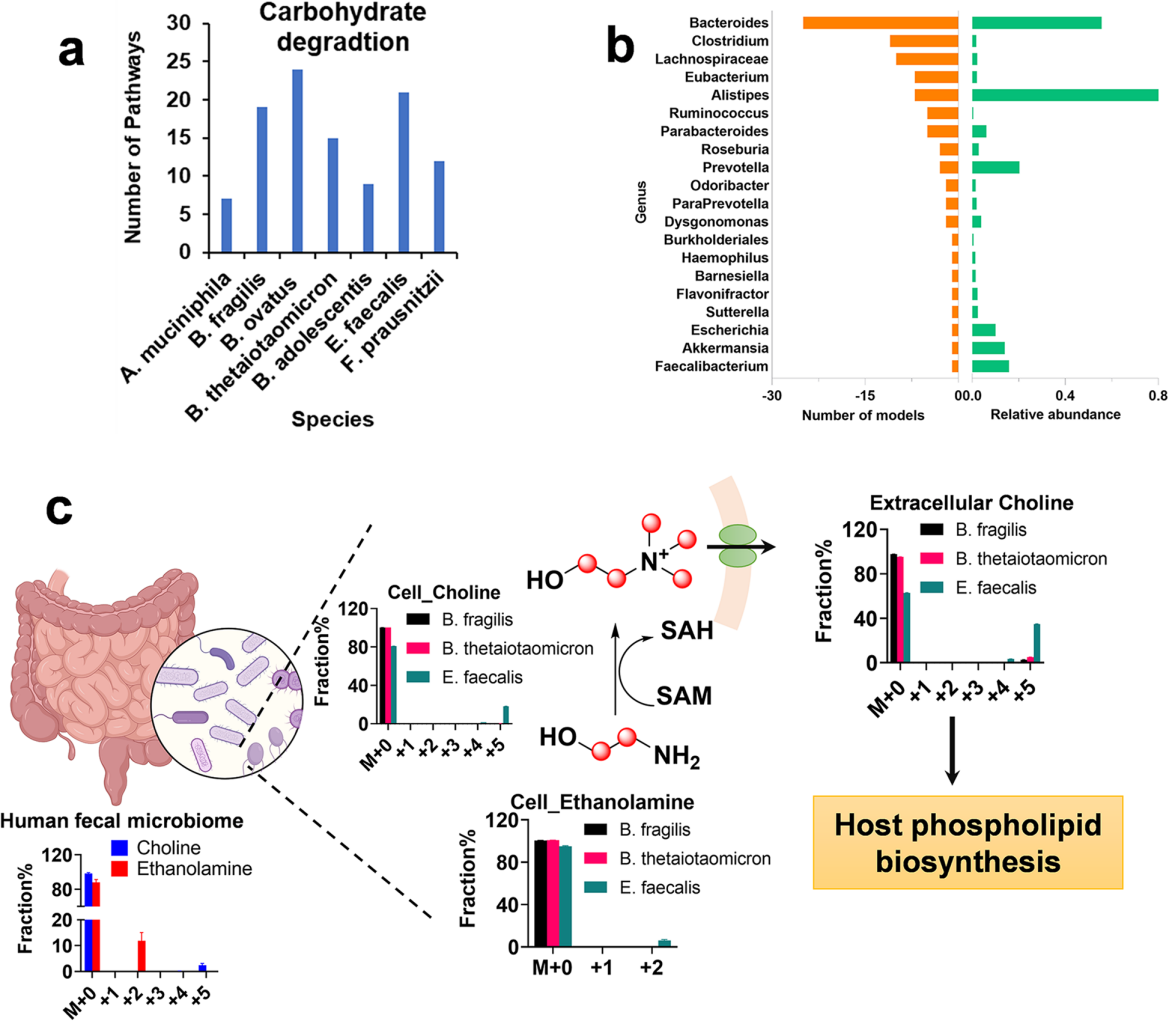
In silico and in vitro identification of gut microbial species that contribute to the degradation of inulin and biosynthesis of ^13^C_5_-choline. **a** Comparative-analysis of metabolic pathways in seven representative strains that are capable of metabolizing carbohydrates. The total number of pathway classes associated with carbohydrate degradation in each species was compared using MetaCyc and calculated using Excel. **b** The biochemical potential of gut microbiome-related metabolic models in the HMP database was examined for the capability to produce choline. The top 20 genera with the highest number of models are shown. **c** The production of ethanolamine and choline in the human fecal microbiome, *B. fragilis*, *B. thetaiotaomicron*, and *E. faecalis*. ^13^C fractional enrichments of choline and its precursor ethanolamine were showed. Detection of ^13^C-labeled choline in culture media indicates that choline synthesized in the gut microbiome can be exported to the extracellular pool and contribute to the host phospholipid biosynthesis. Red circle: ^13^C

To determine the role of gut microbes in degrading [U-^13^C]-inulin to produce ^13^C_5_-choline, we compared the metabolic pathways of seven gut microbes that are known to be carbohydrate degraders using the MetaCyc database. It was found that *B. ovatus*, *B. fragilis*, *B. thetaiotaomicron*, and *E. faecalis* were the bacterial species with the highest number of carbohydrate degradation pathways (Fig. 8a). In addition, inulin degradation was assigned in MetaCyc as a unique pathway in *E. faecalis* compared with the other tested species. Serine, ethanolamine, phosphoethanolamine, and phosphocholine can be used as substrates by bacteria to synthesize choline. We collected enzymes in the choline biosynthesis pathway from the NCBI Reference Sequence Database and compared them with those in the HMP database. The results showed that enzymes related to choline synthesis are widely distributed in the gut microbes, including *Bacteroides*, *Clostridium*, *Alistipes*, *Parabacteroides*, *Lachnospiraceae*, and *Eubacterium*, with *Bacteroides* being the major genus that catalyzes the production of choline taking into considerations of the number of models and relative abundance in the human gut (Fig. 8b).

*B. fragilis* and *B. thetaiotaomicron*, the major *Bateroides* in the human gut [14], as well as *E. faecalis* were selected as representative species and were incubated with [U-^13^C]-inulin separately. In addition, we incubated the human fecal microbiome in vitro with [U-^13^C]-inulin. It was found that ^13^C_5_-choline was the major isotopologue produced by the human fecal microbiome and monocultures of these three microbes (Fig. 8c). Furthermore, fully labeled ^13^C_2_-ethanolamine was detected in the human fecal microbiome and *E. faecalis* incubation samples (Fig. 8c). The ^13^C fractional enrichment of ^13^C_5_-choline in microbial cells was 0.25% for both *B. fragilis* and *B. thetaiotaomicron*, whereas it was 18.1% for *E.* faecalis. The fractional enrichment of ^13^C_2_-ethanolamine, a precursor of ^13^C_5_-choline, was 6.2% in *E. faecalis* and was below the detection limit in *Bateroides* spp. (Fig. 8c). The results suggested a higher activity of *E. faecalis* than *Bateroides* spp. in metabolizing [U-^13^C]-inulin to produce ^13^C-fully labeled choline via the ethanolamine pathway. In the culture media, the ^13^C fractions of ^13^C_5_-choline were 2.7, 5.0, and 34.8% for *B. fragilis* and *B. thetaiotaomicron*, and *E. faecalis*, respectively (Fig. 8c), indicating that newly synthesized choline could be actively transported from the intracellular pool to the extracellular compartment, where it could be absorbed by the enterocytes of the host and used for phospholipid biosynthesis.

## Discussion

### The advantages of SIRM in investigating the gut microbiome-host organ communications

In this investigation, we have established a SIRM method that revealed the metabolic connections between the gut microbiome and host organs, including the liver, brain, skeletal muscle, and systemic circulation. The demonstrated advantages of this approach include the followings: (1) The in vivo metabolome changes observed in this study could be considered as molecular phenotypic representations originating from the gut microbiome, which has been beyond the reach of widely used germ-free mouse studies. This is because [U-^13^C]-inulin, the stable isotopic tracer used in this study, is a polysaccharide subjected to degradation by the gut microbiome rather than absorption by the enterocytes after oral administration; (2) The sampling schedule avoided the time window when directly absorbed mono- and di-polysaccharides could be metabolized. For the time-dependent analysis, the sampling time points were set at 6, 12, and 24 h. This schedule was based on the fact that the GI transit time is approximately 6 h in mice [22]. In addition, [U-^13^C]-inulin contained 12.6% of mono- and di-polysaccharides, which could be absorbed directly without gut microbial degradation and incorporated into the host metabolic pathways. It has been reported that the orally gavaged glucose, fructose, and sucrose can be fully excreted after 3 h in rodents [23]. Williams et al. reported a stable isotope metabolomic study in which C57BL/6 mice were orally gavaged with [U-^13^C]-glucose at 2 g/kg [24]. The ^13^C fractional enrichment in plasma was investigated up to 4 h post-gavage. The ^13^C-glucose concentration peaked at approximately 15 min, with more than 50% of the plasma glucose being fully labeled with ^13^C, and less than 5% of the circulating glucose was fully labeled at 4 h post-gavage. ^13^C fractional enrichment of metabolites showed that the highest labeling for most of the metabolites was observed at 15 min post-gavage, followed by a steady decrease until 4 h. However, citrate and other metabolites associated with the Krebs cycle showed a slower enrichment that peaked at 2 h and decreased thereafter. Taken together, although we cannot rule out the possibility that ^13^C isotopologues derived from ^13^C-monosaccharide or disaccharide impurities still exist at 6 h after ^13^C-inulin administration, their contents could be low, and the interference with ^13^C-inulin metabolism could be minimal. Therefore, the current sampling schedule could on the one hand ensure that the orally gavaged [U-^13^C]-inulin has arrived at the cecum, the major site of gut microbial metabolism in mouse, and on the other hand avoid interferences from the absorbed ^13^C mono- and di-saccharides, which have been completely excreted at 6 h after [U-^13^C]-inulin administration; (3) comprehensive coverage of metabolic networks such that the turnover of lipids, nucleotides, amino acids, and organic acids are observable. We found that the labeling of most of the metabolome in all the biological samples peaked at 6 h after [U-^13^C]-inulin administration; however, the highest labeling of lipids was achieved at 12 h, suggesting a time lag of ^13^C-flux from central carbon metabolism to lipid metabolism in the gut microbiome. Taken together, these advantages represent a major step forward for in vivo holistic studies of host and gut microbiome interorgan metabolic connections. However, there are limitations in the current study. Because of the qualitative nature of the SIRM method, we cannot determine how much metabolites are produced from ^13^C-inulin, and further quantitative studies are warranted to address this important question.

### De novo synthesis of choline in the gut microbiome and contributions to the host choline pool

To the best of our knowledge, this is the first study to address a new role for the gut microbiome in the de novo synthesis of choline, which can be generated from the ethanolamine pathway. Choline is an essential nutrient that functions in multiple physiological pathways and can be broadly divided into 3 categories: (1) as a component of phosphatidylcholine and sphingomyelin, which are critically important for cell membrane lipids and plasma lipoproteins; (2) a precursor of the neurotransmitter acetylcholine; and (3) downstream metabolism of choline produces trimethylamine and trimethylamine N-oxide, which is associated with cardiometabolic diseases. It is well acknowledged that choline can be acquired from the diet and via biosynthesis through the methylation of phosphatidylethanolamine (PE) to form PC in human organs. We detected the ^13^C_5_-fully labeled choline in mouse cecum content after ^13^C-inulin administration. In vitro studies revealed that ^13^C_5_-choline was produced by human fecal microbes incubated with ^13^C-inulin, suggesting a new source of choline derived from gut microbiome biosynthesis. Labeled choline and phospholipids in mouse plasma and tissues further confirmed the contribution of microbe-derived choline to the host lipid metabolism. We found that ^13^C_5_-choline has a longer in vivo residence time in the cecum content and host organs than other labeled polar metabolites, which were mostly eliminated at 12 h (cf. Figure 2, 5). One possible mechanism is that ^13^C_5_-choline can be constantly generated. Hydrolysis of phosphatidylcholine to choline can be catalyzed by phospholipase D enzymes from diverse members of the gut microbiome [25]. Therefore, gut commensals can recycle ^13^C-phosphatidylcholine to produce ^13^C_5_-choline, and choline moiety of lipids returns back to the choline pool. Another possible mechanism is that ^13^C_5_-choline may have a long half-life, and the relatively high abundance of ^13^C_5_-choline at 24 h indicates this as an alternative explanation to the steady production in the gut. It should be noted that although high fractional enrichment of ^13^C_2_-ethanolamine was detected in the cecum content, ^13^C_2_- and ^13^C_4_-choline, which were presumably derived from ^13^C_2_-ethanolamine, were detected at trace levels if any. However, ^13^C_5_-choline (fully labeled) was produced at a relatively high abundance. The mechanism presumably involves steady production of ^13^C_5_-choline from ^13^C-phosphatidylcholine and/or longer half-life of ^13^C_5_-choline than of other choline isotopologues. Further research is needed to understand this interesting phenomenon. Additionally, the importance of microbe-derived choline in human health and disease warrants further investigation.

### Tissue preferences in utilizing ^13^C derived from the gut microbiome

^13^C-Metabolome profiles in different host organs suggested a tissue-specific preference for utilizing ^13^C derived from the gut microbiome. In the brain, the differentially expressed metabolites with a high number of ^13^C labeling (*n* ≥ 3) included GABA, choline, N-acetylaspartate (NAA), lactate, inosine, and N-acetylneuraminate (NeuAc). GABA is a neurotransmitter that can be synthesized in both neurons and gut microbes [26]. Distinct ^13^C enrichment profiles of GABA were observed in different organs. We identified ^13^C_4_ as the major isotopologue for GABA in the mouse cecum content, which was consistent with our previous in vitro study using the mouse fecal microbiome [11]. The major isotopologues of GABA in the liver and brain were ^13^C_1_ and ^13^C_2_, consistent with the ^13^C profile of glutamate, the precursor for GABA. These results suggested that glutamate decarboxylation is the major route of GABA production in host organs, and the gut microbiome could probably use other precursors (i.e., ornithine, putrescine, and arginine [11, 26]) for GABA biosynthesis, which resulted in a fully labeled isotopologue. Notably, although presented at trace levels that the LC–MS peak cannot be integrated, the MS signal of ^13^C_4_-GABA (*m/z* 108.0843) was readily detected in the mouse brain, suggesting the potential contribution of gut microbiome-derived GABA to the brain pool. NAA is the most concentrated metabolite in the mammalian brain and is localized predominantly in neurons. It has been used as a biomarker for investigating neurological and metabolic disorders [27, 28]. The identification of ^13^C-NAA in both the cecum content and brain suggested that the gut microbiome could contribute to the brain pool of NAA through either the absorption of ^13^C-NAA synthesized by the gut bacteria or ^13^C/^12^C-acetylation of ^13^C/^12^C-aspartate in host organs. Lactate has recently been characterized as a signaling molecule in the brain [29]. The ^13^C-lactate fraction profile is similar between brain and plasma, suggesting that circulating ^13^C-lactate could be taken up by the brain via an equilibration mechanism. These results provide direct evidence on gut-brain interactions mediated by metabolites.

In skeletal muscle, the differentially expressed metabolites with a high number of ^13^C labelings (*n* ≥ 2) included lactate, butyrylcarnitine, acetylcarnitine, 3-hydroxybutyrate glutamine, citrulline, and choline. The ^13^C enrichment profile of lactate in skeletal muscle was different from that in other host organs where mixed labeling of lactate was detected, indicating that skeletal muscle cells could either uptake circulating gut microbiome-derived lactate (^13^C_3_-lacate in cecum content) or actively produce ^13^C-lactate from the ^13^C source in situ. A trend toward higher ^13^C-lactate enrichment was observed in male mice at 12 h after ^13^C-inulin administration, suggesting that the skeletal muscle of male mice is more active in utilizing gut microbiome-derived ^13^C to produce ^13^C-lactate. It was reported that athlete running performance could benefit from the gut microbiota, which takes up lactate and disposes of it as propionate [30]. The current study provided new evidence on the “gut-soma lactate shuttle” [31]. We propose that the gut microbiome could supply lactate to skeletal muscle cells by degrading dietary polysaccharides. In addition, this effect was likely be more pronounced in males than in females. Carnitine plays multiple roles in skeletal muscle metabolism, including fatty acid transport and buffering of excess acetyl-CoA in the mitochondria. In skeletal muscle cells, carnitine can be converted to ^13^C_2_-acetylcarnitine and ^13^C_2_/^13^C_4_-butyrylcarnitine by reacting with ^13^C_2_-acetyl-CoA. 3-Hydroxybutyrate [32], citrulline [33], glutamine [34], and choline [35] are important nutrients for skeletal muscle functions, including the regulation of insulin resistance and metabolic homeostasis. Identification of ^13^C-labeled metabolites revealed that the ^13^C flows from the gut microbiome to bioactive metabolites in skeletal muscle, which underlines the close metabolic interactions between the gut microbiota and the skeletal muscle.

### Gut microbe species that degrade inulin and produce choline

*Bacteroides* spp. are notable for displaying broad plasticity for glycan utilization with polysaccharide utilization loci (PUL) and multiple carbohydrate active enzymes [36]. However, the fructan PUL is conserved to varying extents among *Bacteroides* species, corresponding to a range of fructan utilization capability [37]. We identified varied capacities of bacterial species to catalyze the biosynthesis of ^13^C_5_-choline after degrading ^13^C-inulin, with *E. faecalis* being more efficient than *B. fragilis* and *B. thetaiotaomicron*. The results suggested that the polysaccharide of β-(2,1) fructose might be a preferred substrate for *E. faecalis* rather than for *Bacteroides* spp. Moreover, the choline synthesized in *E. faecalis* could be actively transported to extracellular compartments, thus contributing to the host choline pool. The discovery of the metabolic function of *E. faecalis* has implications for human health and disease. It was recently found that *E. faecalis* could affect social behaviors and mediate stress responses in the brain [38]. The current study identified choline as a neurochemical that can serve as a common language between host and gut microbiome. Moreover, choline can be degraded to generate trimethylamine, which is further oxidized in the liver to produce trimethylamine *N*-oxide (TMAO). Elevated plasma TMAO levels have been correlated with cardiovascular diseases [39]. Our results raise the possibility that the gut microbiome may serve to influence the circulating TMAO through choline de novo biosynthesis pathways.

## Conclusions

In the present study, we developed a SIRM-based mapping of the gut microbiome-host metabolic networks. Using ^13^C-inulin as a tracer, we found that the gut microbiome extensively metabolized ^13^C-inulin and produced biochemicals that could be assimilated by the host organs. Choline metabolism, the glutamine-glutamate/GABA cycle, and lactate metabolism were the major ^13^C-labeled pathways in the liver, brain, and skeletal muscle, respectively. In addition, the gut microbiome was shown to play an important role in de novo synthesis of choline, which contributed to the host lipogenesis. By identifying previously unrecognized interconnecting metabolites, our findings enhance the understanding of how a prebiotic such as inulin affects host metabolism through the microbiome, and of the interorgan transported metabolites that connect the gut microbiome with the host organs.

### Supplementary Information

**Supplementary Material 1.**

**Supplementary Material 2.**

**Supplementary Material 3.**

**Supplementary Material 4.**

**Supplementary Material 5.**

**Supplementary Material 6.**

**Supplementary Material 7.**

**Supplementary Material 8.**

**Supplementary Material 9.**

**Supplementary Material 10.**

**Supplementary Material 11.**

## Data Availability

All the data generated or analyzed during this study are included in this published article [and its supplementary information files]. The raw LC‒MS data can be requested by contacting the corresponding author Pan Deng (pandeng@suda.edu.cn).

## Abbreviations

BCAAs: Branched-chain amino acids
DP: Degree of polymerization
GABA: γ-Aminobutyric acid
HMP: Human microbiome project
LC-HRMS: Liquid chromatography-high resolution mass spectrometry
LPC: Lysophosphatidylcholine
MEBA: Multivariate empirical Bayes time-series analysis
MTBE: Methyl tertiary-butyl ether
NAA: N-acetylaspartate
PC: Phosphatidylcholine
PCA: Principal component analysis
PE: Phosphatidylethanolamine
PPP: Pentose phosphate pathway
PUL: Polysaccharide utilization locus
qMSEA: Quantitative metabolite set enrichment analysis
SAM: S-adenosylmethionine
SCFAs: Short-chain fatty acids
SIRM: Stable isotope resolved metabolomics
TMAO: Trimethylamine *N*-oxide
