# Supplementary material for: 13C-Stable isotope resolved metabolomics uncovers dynamic biochemical landscape of gut microbiome-host organ communications in mice
Source: Microbiome. 2024 May 15;12:90. doi: 10.1186/s40168-024-01808-x (PMC11094917; doi:10.1186/s40168-024-01808-x)
Supplement: Supplementary file 12 — Supplementary Material 11. [file 40168_2024_1808_MOESM11_ESM.docx]

**Supplementary Information**

**^13^C-Stable isotope resolved metabolomics uncovers dynamic biochemical landscape of gut microbiome-host organ communications in mice**

Xia Xiao^1^, Yixuan Zhou^1^, Xinwei Li^1^, Jing Jin^1^, Jerika Durham^2,3^, Zifan Ye^4^, Yipeng Wang^4^, Bernhard Hennig^2,5*^, Pan Deng^1*^

^1^ Jiangsu Key Laboratory of Neuropsychiatric Diseases and College of Pharmaceutical Sciences, Soochow University, Suzhou, China

^2^ Superfund Research Center, University of Kentucky, Lexington, KY, USA

^3^ Department of Toxicology and Cancer Biology, College of Medicine, University of Kentucky, Lexington, KY, USA

^4^ Department of Biopharmaceutical Sciences, College of Pharmaceutical Sciences, Soochow University, Suzhou, China

^5^ Department of Animal and Food Sciences, Martin-Gatton College of Agriculture, Food and Environment, University of Kentucky, Lexington, KY, USA

**List of Items**

Supplementary Fig. 1

HPAEC-PAD chromatogram of [U-^13^C]-inulin chemical standard.

Supplementary Fig. 2

LC-HRMS analysis of biochemical unknown_5 and its isotopologues.

Supplementary Fig. 3

^13^C labeling of glutamate detected in the brain.

Supplementary Fig. 4

LC-HRMS analysis of the ^13^C isotopologues of PC34:2 and LPC16:0 detected in the plasma.


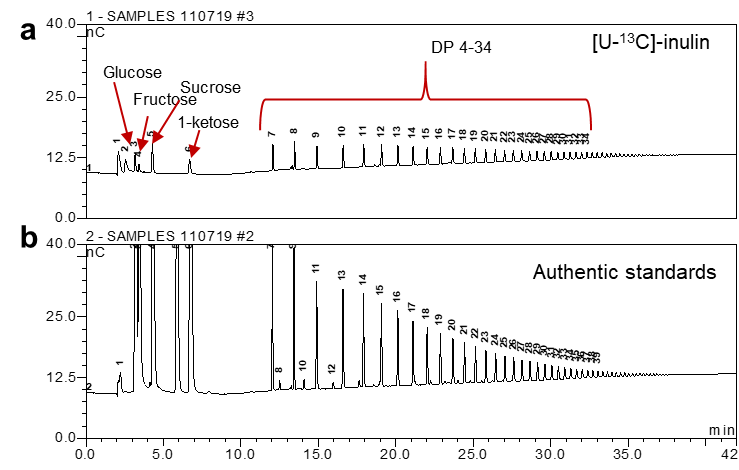


**Supplementary Fig. 1. High-Performance Anion Exchange Chromatography-pulsed amperometric detection (HPAEC-PAD) chromatogram of [U-^13^C]-inulin.** The degree of polymerization of [U-^13^C]-inulin was analyzed by using a HPAEC-PAD method. **a**: [U-13C]-inulin; **b**: mono/polysaccharides standards including glucose, fructose, sucrose (DP 2), 1-ketose (DP 3), and inulin (Raftiline, DP 3 to 60).


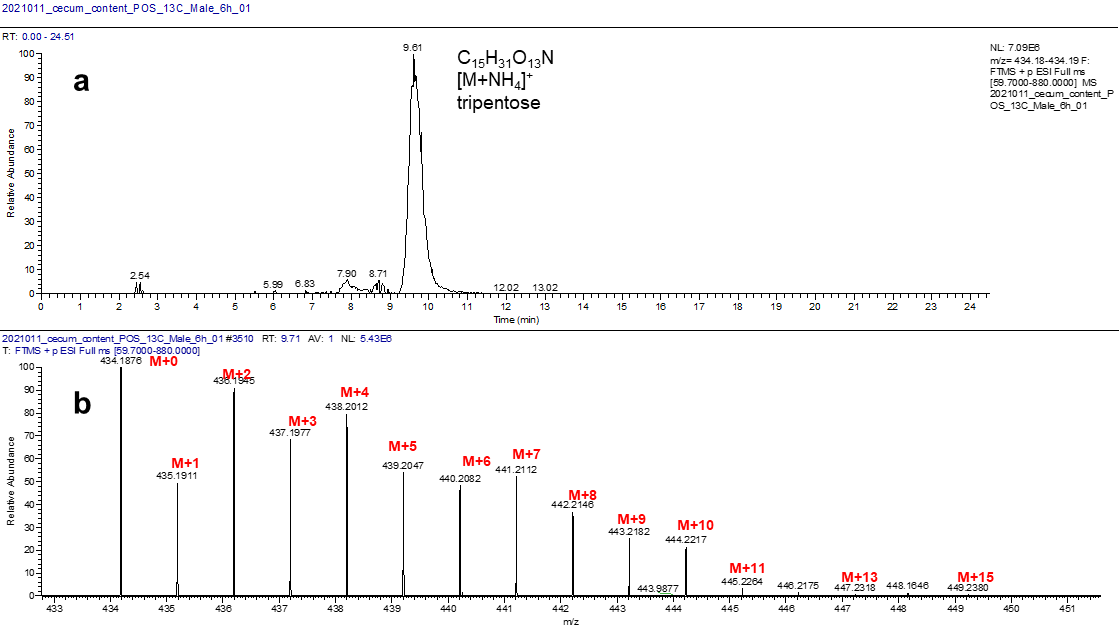


**Supplementary Fig. 2. LC-HRMS analysis of biochemical unknown_5 and its isotopologues in the cecum content.** A biochemical (named as unkown_5, *m/z* 434.1875) and its isotopologues was detected in the cecum content at 6 h after [U-^13^C]-inulin administration by using untargeted LC-HRMS. **a**) Extracted ion chromatogram of the biochemical unknown_5; **b**) Full scan mass spectrum of the biochemical shows the precursor ions of this biochemical and its isotopologues with the number of ^13^C ranging from 1 to 15.


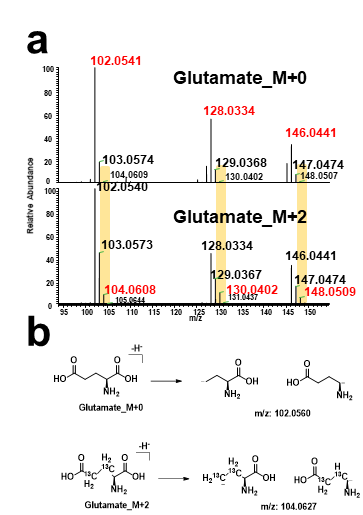


**Supplementary Fig. 3. ^13^C labeling of glutamate in the brain**. **a**) Full scan mass spectra of glutamate (Glutamate_M+0) and its major isotopologue (Glutamate_M+2) in the brain sample. Characteristic fragments at *m/z* 104.0608 and 130.0402 were detected for Glutamate_M+2, which are 2 Da higher than the corresponding fragments for non-labeled glutamate. **b**) Proposed fragment pathways for Glutamate_M+0 and Glutamate_M+2. Based on the fragments, the ^13^C labeling sites were proposed at non-terminal carbons.


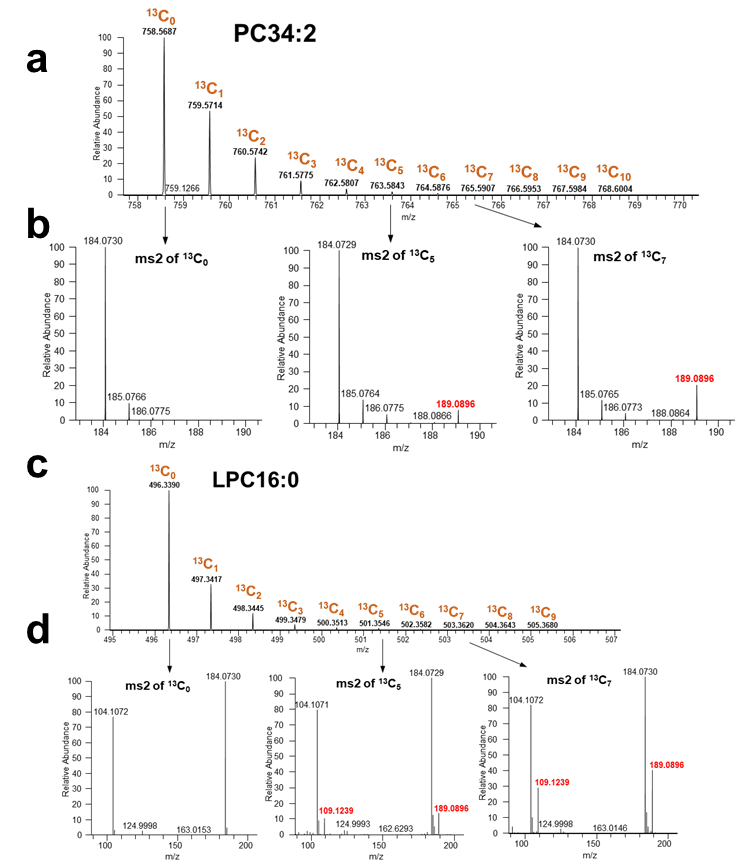


**Supplementary Fig. 4. LC-HRMS analysis of the ^13^C isotopologues of PC34:2 and LPC16:0 in the plasma**. **a**) Full scan mass spectrum of PC34:2 and its isotopologues in the plasma; b) ms2 fragment of precursor ions at *m/z* 758.5687, 763.5843, and 765.5907, corresponding to PC34:2, ^13^C_5_-PC34:2 and ^13^C_7_-PC34:2. The detection of characteristic fragment at *m/z* 189.0896 indicates that the labeling happens on the choline head group; c) full scan mass spectrum of LPC16:0 and its isotopologues in the plasma; **b**) ms2 fragment of precursor ions at *m/z* 496.3390, 501.3546, and 503.3620, corresponding to LPC16:0, ^13^C_5_-LPC16:0 and ^13^C_7_-LPC16:0. The detection of characteristic fragments at *m/z* 189.0896 and 109.1239 indicates that the labeling happens on the choline head group.
